# 2-Oxo-2,3-dihydro-1*H*-imidazo[1,2-*a*]pyridinium iodide

**DOI:** 10.1107/S1600536810004976

**Published:** 2010-02-13

**Authors:** Jinling Miao, Jisheng Guo, Chunhua Hu, Daqi Wang, Yong Nie

**Affiliations:** a School of Chemistry and Chemical Engineering, University of Jinan, Jinan 250022, People’s Republic of China; b Department of Chemistry, New York University, 100 Washington Square East, New York, NY 10003-6688, USA; c College of Chemistry and Chemical Engineering, Liaocheng University, Liaocheng 252059, People’s Republic of China

## Abstract

In the title compound, C_7_H_7_N_2_O^+^·I^−^, the carbonyl C and O atoms of the cation and the iodide ion are situated on mirror planes. The mean plane of the imidazo[1,2-*d*]pyridinium cation is perpendicular to the mirror plane as a consequence of the disorder of the cation over two opposite orientations of equal occupancy. In the crystal, N—H⋯I interactions are present.

## Related literature

For the synthesis of imidazo[1,2-*a*]pyridinium chloride or bromide, see: Newton *et al.* (1984 ▶); Baumann *et al.* (1986 ▶). For the derivatization of imidazo[1,2-*a*]pyridinium and related structures, see: Plutecka *et al.* (2006 ▶); Hoffmann *et al.* (2005 ▶); Qiao *et al.* (2006 ▶).
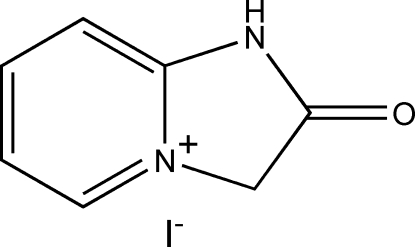


## Experimental

### 

#### Crystal data

C_7_H_7_N_2_O^+^·I^−^
*M* *_r_* = 262.05
Orthorhombic, 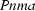

*a* = 14.597 (2) Å
*b* = 8.2044 (18) Å
*c* = 7.0926 (15) Å
*V* = 849.4 (3) Å^3^
*Z* = 4
Mo *K*α radiation
μ = 3.71 mm^−1^
*T* = 298 K
0.48 × 0.45 × 0.23 mm

#### Data collection

Bruker SMART 1000 CCD area-detector diffractometer
Absorption correction: multi-scan (*SADABS*; Bruker, 2001 ▶) *T*_min_ = 0.269, *T*_max_ = 0.482
3631 measured reflections
806 independent reflections
691 reflections with *I* > 2σ(*I*)
*R*_int_ = 0.064

#### Refinement

*R*[*F* ^2^ > 2σ(*F* ^2^)] = 0.036
*wR*(*F* ^2^) = 0.103
*S* = 1.05
806 reflections
73 parameters
24 restraints
H-atom parameters constrained
Δρ_max_ = 0.70 e Å^−3^
Δρ_min_ = −0.93 e Å^−3^

### 

Data collection: *SMART* (Bruker, 2001 ▶); cell refinement: *SAINT* (Bruker, 2001 ▶); data reduction: *SAINT*; program(s) used to solve structure: *SHELXS97* (Sheldrick, 2008 ▶); program(s) used to refine structure: *SHELXL97* (Sheldrick, 2008 ▶); molecular graphics: *SHELXTL* (Sheldrick, 2008 ▶); software used to prepare material for publication: *SHELXTL*.

## Supplementary Material

Crystal structure: contains datablocks I, global. DOI: 10.1107/S1600536810004976/cv2672sup1.cif

Structure factors: contains datablocks I. DOI: 10.1107/S1600536810004976/cv2672Isup2.hkl

Additional supplementary materials:  crystallographic information; 3D view; checkCIF report

## Figures and Tables

**Table 1:** Hydrogen-bond geometry (Å, °)

*D*—H⋯*A*	*D*—H	H⋯*A*	*D*⋯*A*	*D*—H⋯*A*
N1—H2*A*⋯I1^i^	1.03	2.85	3.80 (2)	153

Symmetry code: (i) 
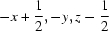
.

**Fig. 1.**
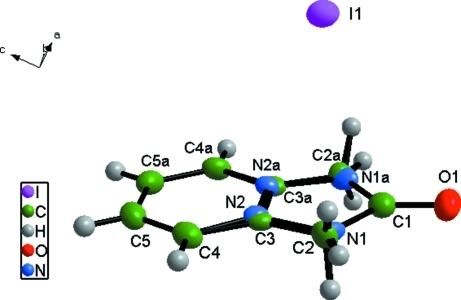
The molecular structure, with atom labels and 25% probability displacement ellipsoids [symmetry code: (a) x, 1/2 - y, z].

## supplementary crystallographic information

### Comment

Imidazo[1,2-*a*]pyridine derivatives have been investigated as important intermediates in organic synthesis and useful agents in medicinal chemistry. Imidazo[1,2-*a*]pyridinium chloride or bromide is accessible from the reaction of alkyl haloacetate with 2-aminopyridine compounds (Newton *et al.*, 1984; Baumann *et al.*, 1986), and can be further derivatised (Plutecka *et al.*, 2006; Hoffmann *et al.*, 2005). The reaction of 2-aminopyridine and chloroacetic acid under basic condition gave rise to, after acidification, 3,3-bis(carboxymethyl) imidazo[1,2-*a*]pyridine-2-one (Qiao *et al.*, 2006). Here we report on the synthesis and structure of the title compound (I), which was obtained from the reaction of iodoacetic acid with 2-aminopyridine under basic condition.

The structure of (I) (Fig. 1) consists of imidazo[1,2-*a*]pyridinium cations and iodide anions. In the cation, the six-membered and five-membered rings are coplanar with a dihedral angle of 0.48°. However, the four C/N atoms in the ring system (Fig. 1) are found to be disordered. The structure may be seen as two molecules being in one crystallographic position, with an occupancy of 0.5 for each C/N atom involved. Thus, in one molecule the five-membered ring is N2/C2/C1/N1a/C3a, and in another molecule - C3/N1/C1/C2a/N2a.

### Experimental

A mixture of 2-aminopyridine (1.132 g, 0.012 mol), ICH_2_COOH (5.592 g, 0.030 mol) and Na_2_CO_3_ (2.549 g, 0.024 mol) was placed in 60 ml of distilled water. After the evolution of bubbles was over, the mixture of was heated at reflux for 6 h, while the pH was adjusted to 8–9 using aqueous NaOH (0.1 mol/l) solution, at a time interval of 0.5 h. The resulting deep red solution was cooled to room temperature and acidified with hydrochloric acid till pH 2–3 (during which some red solid was formed, but could be dissolved on warming to 40°C). On standing still at room temperature, deep red crystals were grown after one month. IR (KBr): 3465, 3076, 1751, 1650, 1511, 1330, 1185, 792, 608 cm^-1^.

### Refinement

All H atoms were positioned geometrically and refined using a riding model with C—H = 0.93–0.97 Å, N—H = 0.86 Å, *U*_iso_(H) = 1.2*U*_eq_(C,N).

### Crystal data

**Table:**

C_7_H_7_N_2_O^+^·I^−^	*D*_x_ = 2.049 Mg m^−^^3^
*M**_r_* = 262.05	Mo *K*α radiation, λ = 0.71073 Å
Orthorhombic, *P**n**m**a*	Cell parameters from 1914 reflections
*a* = 14.597 (2) Å	θ = 2.5–27.2°
*b* = 8.2044 (18) Å	µ = 3.71 mm^−^^1^
*c* = 7.0926 (15) Å	*T* = 298 K
*V* = 849.4 (3) Å^3^	Block, red
*Z* = 4	0.48 × 0.45 × 0.23 mm
*F*(000) = 496	

### Data collection

**Table:**

Bruker SMART 1000 CCD area-detector diffractometer	806 independent reflections
Radiation source: fine-focus sealed tube	691 reflections with *I* > 2σ(*I*)
graphite	*R*_int_ = 0.064
ω scans	θ_max_ = 25.0°, θ_min_ = 2.8°
Absorption correction: multi-scan (*SADABS*; Bruker, 2001)	*h* = −17→13
*T*_min_ = 0.269, *T*_max_ = 0.482	*k* = −9→9
3631 measured reflections	*l* = −5→8

### Refinement

**Table:**

Refinement on *F*^2^	Primary atom site location: structure-invariant direct methods
Least-squares matrix: full	Secondary atom site location: difference Fourier map
*R*[*F*^2^ > 2σ(*F*^2^)] = 0.036	Hydrogen site location: inferred from neighbouring sites
*wR*(*F*^2^) = 0.103	H-atom parameters constrained
*S* = 1.05	*w* = 1/[σ^2^(*F*_o_^2^) + (0.0619*P*)^2^ + 0.9786*P*] where *P* = (*F*_o_^2^ + 2*F*_c_^2^)/3
806 reflections	(Δ/σ)_max_ < 0.001
73 parameters	Δρ_max_ = 0.70 e Å^−^^3^
24 restraints	Δρ_min_ = −0.93 e Å^−^^3^

### Special details

**Table:**

Geometry. All e.s.d.'s (except the e.s.d. in the dihedral angle between two l.s. planes) are estimated using the full covariance matrix. The cell e.s.d.'s are taken into account individually in the estimation of e.s.d.'s in distances, angles and torsion angles; correlations between e.s.d.'s in cell parameters are only used when they are defined by crystal symmetry. An approximate (isotropic) treatment of cell e.s.d.'s is used for estimating e.s.d.'s involving l.s. planes.

### Fractional atomic coordinates and isotropic or equivalent isotropic displacement parameters (Å^2^)

**Table:**

	*x*	*y*	*z*	*U*_iso_*/*U*_eq_	Occ. (<1)
I1	0.41289 (4)	0.2500	0.91066 (7)	0.0537 (3)	
C4	0.1109 (3)	0.0822 (7)	0.9883 (8)	0.0519 (13)	
H4	0.1112	−0.0311	0.9844	0.062*	
C5	0.0758 (4)	0.1656 (8)	1.1372 (9)	0.0551 (14)	
H5	0.0517	0.1091	1.2395	0.066*	
O1	0.2504 (5)	0.2500	0.4103 (7)	0.0724 (19)	
C1	0.2120 (6)	0.2500	0.5605 (11)	0.053 (2)	
C2	0.184 (3)	0.103 (3)	0.674 (4)	0.050 (9)	0.50
H2A	0.1384	0.0386	0.6073	0.060*	0.50
H2B	0.2363	0.0342	0.7024	0.060*	0.50
N2	0.146 (4)	0.174 (3)	0.846 (4)	0.039 (8)	0.50
N1	0.186 (2)	0.1164 (19)	0.666 (3)	0.049 (7)	0.50
H1	0.1935	0.0170	0.6309	0.058*	0.50
C3	0.146 (4)	0.161 (4)	0.831 (5)	0.037 (8)	0.50

### Atomic displacement parameters (Å^2^)

**Table:**

	*U*^11^	*U*^22^	*U*^33^	*U*^12^	*U*^13^	*U*^23^
I1	0.0550 (4)	0.0434 (4)	0.0627 (4)	0.000	−0.0103 (2)	0.000
C4	0.044 (3)	0.044 (3)	0.068 (3)	0.002 (2)	−0.003 (3)	0.009 (3)
C5	0.046 (3)	0.064 (4)	0.056 (3)	0.000 (2)	−0.001 (2)	0.013 (3)
O1	0.070 (4)	0.099 (5)	0.049 (3)	0.000	0.003 (3)	0.000
C1	0.047 (5)	0.058 (5)	0.053 (5)	0.000	−0.004 (4)	0.000
C2	0.054 (13)	0.039 (10)	0.057 (12)	−0.006 (8)	0.009 (8)	0.005 (8)
N2	0.032 (10)	0.040 (9)	0.047 (9)	0.004 (7)	−0.003 (7)	−0.002 (6)
N1	0.047 (11)	0.042 (9)	0.058 (11)	0.003 (8)	−0.016 (8)	−0.019 (7)
C3	0.030 (11)	0.034 (10)	0.048 (10)	−0.002 (6)	−0.010 (7)	−0.006 (6)

### Geometric parameters (Å, °)

**Table:**

C4—N2	1.357 (9)	C1—N1^i^	1.381 (9)
C4—C5	1.358 (9)	C1—C2	1.509 (10)
C4—C3	1.387 (9)	C1—C2^i^	1.509 (10)
C4—H4	0.9300	C2—N2	1.461 (10)
C5—C5^i^	1.386 (14)	C2—H2A	0.9700
C5—H5	0.9300	C2—H2B	0.9700
O1—C1	1.204 (9)	N1—C3	1.360 (10)
C1—N1	1.381 (9)	N1—H1	0.8600
			
N2—C4—C5	116.2 (14)	O1—C1—C2^i^	126.8 (12)
N2—C4—C3	6(3)	N1—C1—C2^i^	105.8 (7)
C5—C4—C3	121.9 (16)	N1^i^—C1—C2^i^	1(3)
N2—C4—H4	121.9	C2—C1—C2^i^	106 (2)
C5—C4—H4	121.9	N2—C2—C1	103.3 (11)
C3—C4—H4	116.2	N2—C2—H2A	111.1
C4—C5—C5^i^	120.2 (4)	C1—C2—H2A	111.1
C4—C5—H5	119.9	N2—C2—H2B	111.1
C5^i^—C5—H5	119.9	C1—C2—H2B	111.1
O1—C1—N1	127.5 (10)	H2A—C2—H2B	109.1
O1—C1—N1^i^	127.5 (10)	C4—N2—C2	122.9 (19)
N1—C1—N1^i^	105 (2)	C3—N1—C1	111.7 (10)
O1—C1—C2	126.8 (12)	C3—N1—H1	124.1
N1—C1—C2	1(3)	C1—N1—H1	124.1
N1^i^—C1—C2	105.8 (7)	N1—C3—C4	136 (2)

Symmetry codes: (i) *x*, −*y*+1/2, *z*.

### Hydrogen-bond geometry (Å, °)

**Table:**

*D*—H···*A*	*D*—H	H···*A*	*D*···*A*	*D*—H···*A*
N1—H2A···I1^ii^	1.03	2.85	3.80 (2)	153

Symmetry codes: (ii) −*x*+1/2, −*y*, *z*−1/2.
